# Cancer immunoediting in the melanoma tumor microenvironment: from immune elimination to therapeutic resistance

**DOI:** 10.3389/fimmu.2026.1946627

**Published:** 2026-09-17

**Authors:** Xiaojun Yan, Qian Zhang, Zeping Chen, Haiyan Zhou

**Affiliations:** 1 Department of Dermatology, West China School of Public Health and West China Fourth Hospital, Sichuan University, Chengdu, China; 2 Department of Anaesthesiology, Chongqing Rongchang District People’s Hospital, Chongqing, China; 3 Department of Traditional Chinese Medicine, Chongqing Rongchang District People's Hospital, Chongqing, China

**Keywords:** antitumor immunity, cancer immunoediting, immune checkpoint inhibitors, immune escape, melanoma, tumor microenvironment

## Abstract

Tumor immunoediting is a dynamic process of interaction between the host immune system and tumor cells and encompasses three sequential phases: elimination, equilibrium, and escape. It plays a central regulatory role in melanoma initiation, progression, and therapeutic response. Currently, increasing attention has been directed toward the complexity of the melanoma tumor microenvironment, in which functional dysregulation of immune cell subsets, imbalance of cytokine networks, and alterations in the metabolic milieu collectively drive the process of immunoediting. However, most existing studies have focused on individual phases or isolated factors, and a comprehensive understanding of the entire immunoediting process and its systematic associations with tumor heterogeneity and resistance to immunotherapy remains insufficient. This review summarizes recent advances in tumor immunoediting within the melanoma tumor microenvironment, with particular emphasis on the regulatory mechanisms through which individual components of the immune microenvironment influence different phases of immunoediting, providing a theoretical basis for optimizing melanoma immunotherapy and identifying novel therapeutic targets.

## 1 Introduction

Melanoma is the most aggressive form of skin cancer, with a rising global incidence and poor prognosis in advanced-stage disease (1). As a highly immunogenic malignancy, melanoma develops within a complex tumor immune microenvironment (TIME) composed of heterogeneous immune and stromal cells and diverse signaling networks that critically influence tumor progression and therapeutic response (2). Immune checkpoint inhibitors targeting PD-1/PD-L1 and CTLA-4 have substantially improved outcomes in advanced melanoma, yet primary and acquired resistance remain major clinical challenges (3). Tumor immunoediting provides an important framework for understanding this process, describing the dynamic evolution of tumor–immune interactions through three phases: elimination, equilibrium, and escape (4). During this progression, immune pressure eliminates susceptible tumor cells while selecting resistant clones capable of reducing antigenicity, suppressing immune responses, and ultimately evading immune surveillance.

The tumor microenvironment is the principal site where immunoediting occurs and is dynamically shaped by cancer-associated fibroblasts, tumor-associated macrophages, tumor-infiltrating lymphocytes, dendritic cells, cytokine networks, and metabolic alterations (5, 6). Metabolic reprogramming, including enhanced cholesterol biosynthesis, can further restrict CD8^+^ T-cell infiltration and activation, thereby facilitating immune escape (7). Recent advances in single-cell sequencing and spatial transcriptomics have enabled high-resolution characterization of melanoma cellular heterogeneity and intercellular communication, revealing previously unrecognized regulatory programs within CAF and T-cell populations (8). TME-associated molecular signatures, including PTPRC- and AURKA-related models, have also shown potential for predicting prognosis and immunotherapy response (9, 10). This review therefore summarizes melanoma immunoediting across the elimination, equilibrium, and escape phases, with particular emphasis on microenvironmental remodeling, tumor heterogeneity, immune resistance, and their therapeutic implications.

## 2 Initiation of immunoediting and the elimination phase in the melanoma tumor microenvironment

### 2.1 Role of innate immune responses in the elimination phase of melanoma

Natural killer (NK) cells constitute a first line of innate defense during the elimination phase of melanoma immunoediting. Through NKG2D, NK cells recognize stress-induced ligands such as MICA and MICB on melanoma cells and mediate MHC-independent cytotoxicity, allowing rapid elimination of tumor cells that have downregulated MHC class I to evade T-cell recognition (11). NK cells also shape adaptive immunity by secreting IFN-γ, and their infiltration within the melanoma tumor microenvironment is associated with patient prognosis (12). Dendritic cells (DCs) bridge innate and adaptive immunity by capturing apoptotic or necrotic melanoma cells, processing tumor-associated antigens, and cross-presenting them on MHC class I molecules to prime naïve CD8^+^ T cells (13). Among DC subsets, CD103^+^ conventional type 1 DCs exhibit particularly strong cross-presenting capacity and are essential for initiating melanoma-specific CD8^+^ T-cell responses (14). Type I IFN signaling further supports this process. Tumor-derived cytosolic DNA activates the cGAS–STING pathway, inducing IFN-α/β production, enhancing antigen presentation, and promoting T-cell priming (15). However, melanoma cells may suppress STING signaling through epigenetic silencing or protein degradation, thereby weakening early antitumor immunity (16). Macrophages also contribute to tumor elimination by adopting M1-like phenotypes. These cells kill tumor cells through ROS and NO production and secrete TNF-α and IL-12 to activate NK- and T-cell responses (17–19). However, macrophage plasticity enables later reprogramming toward an M2-like phenotype under tumor-derived IL-10 and TGF-β, facilitating immune evasion and melanoma progression (20, 21). This transition from an M1-like to an M2-like state represents an important turning point in melanoma immunoediting.

### 2.2 Establishment and effector mechanisms of adaptive antitumor immunity

CD8^+^ T cells are the principal adaptive effector cells responsible for melanoma elimination. They recognize melanoma-associated antigens, including MART-1, gp100, and NY-ESO-1, presented by MHC class I molecules through their TCRs (22). Once activated, CD8^+^ T cells kill melanoma cells mainly through perforin/granzyme-mediated cytotoxicity and FasL–Fas-dependent apoptosis (23). Their abundance and functional state are therefore closely associated with prognosis and therapeutic responsiveness in melanoma (24). CD4^+^ helper T cells further support antitumor immunity, particularly Th1 cells, which secrete IFN-γ and IL-2 to promote CD8^+^ T-cell activation, proliferation, survival, and cytotoxicity (25). IFN-γ also enhances antigen presentation by increasing MHC expression on tumor cells and antigen-presenting cells. In addition, Tfh cells may support B-cell responses and antibody-dependent cellular cytotoxicity. Impaired CD4^+^ T-cell function can therefore weaken the overall antitumor immune response (26). Efficient adaptive immunity also depends on chemokine-mediated T-cell trafficking. The CXCL9/CXCL10–CXCR3 axis is a key pathway directing effector T cells into melanoma lesions (27). High CXCL9/CXCL10 expression is associated with increased CD8^+^ T-cell infiltration and improved responses to immune checkpoint blockade, especially anti-PD-1 therapy (28). However, melanoma cells can evade adaptive immune elimination by downregulating MHC class I, acquiring β2-microglobulin mutations, or upregulating inhibitory molecules such as PD-L1 (29–31). The early emergence of these immune-evasion mechanisms allows a subset of melanoma cells to survive immune-mediated elimination and transition into the equilibrium phase of immunoediting, thereby laying the foundation for subsequent immune escape and tumor progression.

## 3 The equilibrium phase of immunoediting and immune remodeling in the melanoma microenvironment

### 3.1 Immune pressure and tumor dormancy during the equilibrium phase

During the equilibrium phase of immunoediting, the immune system is unable to completely eradicate tumor cells but continues to restrain their growth through persistent immune surveillance, forcing residual tumor cells into a reversible state of dormancy. This state is not entirely static but rather reflects a dynamic balance between tumor-cell proliferation and cell death. The equilibrium phase may persist for years or even decades and represents a critical period of prolonged interaction between the tumor and host immune system (32, 33). In a RET transgenic mouse model of melanoma, dormant tumor cells accumulating in the bone marrow, characterized as CD133^+^TRP-2^+^ cells, exhibited low levels of the proliferation marker Ki67 together with altered expression of cell-cycle regulators such as p16 and p27, supporting the existence of a dormant phenotype (34). IFN-γ exerts a dual role during this phase. On the one hand, it sustains antitumor immunity by promoting the activity of cytotoxic T lymphocytes and NK cells. On the other hand, persistent IFN-γ signaling imposes strong selective pressure on tumor cells, favoring the emergence of genetic and epigenetic alterations that reduce tumor immunogenicity and increase resistance to immune-mediated destruction (35–37). Single-cell transcriptomic studies have further suggested that early features of T-cell exhaustion may already emerge during prolonged tumor–immune interactions, including progressive upregulation of inhibitory receptors and exhaustion-associated transcriptional programs such as PD-1 and TOX. At this stage, however, T-cell dysfunction may remain at least partially reversible (38). In parallel, melanoma cells can progressively establish an immunosuppressive microenvironment by increasing PD-L1 expression and promoting the recruitment of Tregs and myeloid-derived suppressor cells (MDSCs), thereby creating conditions that facilitate subsequent immune escape (39).

### 3.2 Establishment of an immunosuppressive network in the tumor microenvironment

During the equilibrium phase, a complex immunosuppressive network is progressively established within the melanoma tumor microenvironment, in which regulatory T cells play a central role. Tregs accumulate within melanoma lesions and suppress antitumor immunity through multiple mechanisms, including secretion of the inhibitory cytokines IL-10 and TGF-β and consumption of local IL-2, thereby restricting the activation, proliferation, and effector functions of conventional T cells (40, 41). Studies have shown that targeting sphingosine kinase 1 (SK1) can reduce Treg infiltration and enhance the efficacy of immune checkpoint blockade, highlighting the therapeutic relevance of Treg-associated immunosuppression in melanoma (42). MDSCs represent another major component of this suppressive network. Through high expression of arginase-1 and inducible nitric oxide synthase, MDSCs perturb local amino acid availability and generate immunosuppressive metabolites, thereby impairing T-cell metabolic fitness, proliferation, and effector function (43). Tumor-associated macrophages (TAMs) also undergo progressive functional reprogramming toward an M2-like, tumor-supportive phenotype. These cells produce high levels of IL-10, TGF-β, and VEGF, thereby suppressing Th1-type antitumor immunity while promoting angiogenesis and tumor progression (21, 44). For example, melanoma-derived exosomal miR-125b-5p has been reported to promote macrophage reprogramming toward a tumor-supportive phenotype through modulation of lysosomal acid lipase A (LIPA)-associated pathways (45). Cancer-associated fibroblasts further reinforce immune suppression by remodeling the extracellular matrix. Increased collagen deposition and accumulation of other matrix components can generate a physical barrier that restricts T-cell penetration into tumor nests. In addition, CAF-derived chemokines such as CXCL12 can contribute to the spatial exclusion of effector T cells from tumor-cell-rich regions (46). Collectively, Tregs, MDSCs, TAMs, and CAFs act in concert to progressively transform the melanoma microenvironment from an immune-controlled state into an increasingly immunosuppressive ecosystem, thereby facilitating the transition from equilibrium to immune escape.

### 3.3 Adaptive changes in melanoma cells during immunoediting

Persistent immune selection during the equilibrium phase drives adaptive changes in melanoma cells and contributes to the progressive development of intratumoral heterogeneity, which represents a fundamental feature of tumor immunoediting (47). Melanoma cells can adapt to sustained immune pressure through multiple mechanisms, including defects in MHC class I or II expression, alterations in antigen-processing and presentation machinery such as TAP1/2 and β2-microglobulin, and loss of immunogenic tumor antigens or neoantigens, collectively reducing their visibility to the immune system (48, 49). HLA-DRB1 expression, for example, has been associated with prognosis and immune activity within the melanoma microenvironment, whereas reduced HLA class II expression may contribute to impaired recognition by CD4^+^ T cells (50). Melanoma cells may also exploit oncogenic signaling pathways to enhance immune resistance. Constitutive activation of MAPK and PI3K-AKT signaling can promote an immunosuppressive phenotype, including increased expression of PD-L1 and reduced sensitivity to immune-mediated killing (51, 52). Moreover, therapeutic perturbation of MAPK signaling can induce broader phenotypic and mechanical adaptations. MAPK pathway inhibition has been shown to trigger mechanotransduction-associated reprogramming through YAP/MRTF-dependent feedback mechanisms, resulting in increased tumor stiffness and the emergence of treatment-resistant states (53). Epigenetic remodeling represents an additional layer of adaptation during immunoediting. Altered DNA methylation and chromatin regulation can silence genes involved in antigen presentation, interferon responsiveness, and immune-cell recruitment, thereby enabling melanoma cells to withstand persistent immune pressure (54, 55). Disruption of the IFN-γ–JAK–STAT axis, whether through genetic or epigenetic mechanisms, can further reduce tumor-cell sensitivity to immune effector signals. Consequently, immunoedited melanoma cells may acquire a phenotype of active immune resistance rather than simple immune ignorance (56). These adaptive changes illustrate how prolonged immune selection reshapes the tumor-cell population and establishes a progressively more heterogeneous and immune-resistant reservoir that ultimately facilitates transition into the escape phase (**Figure 1**).

**Figure 1:**
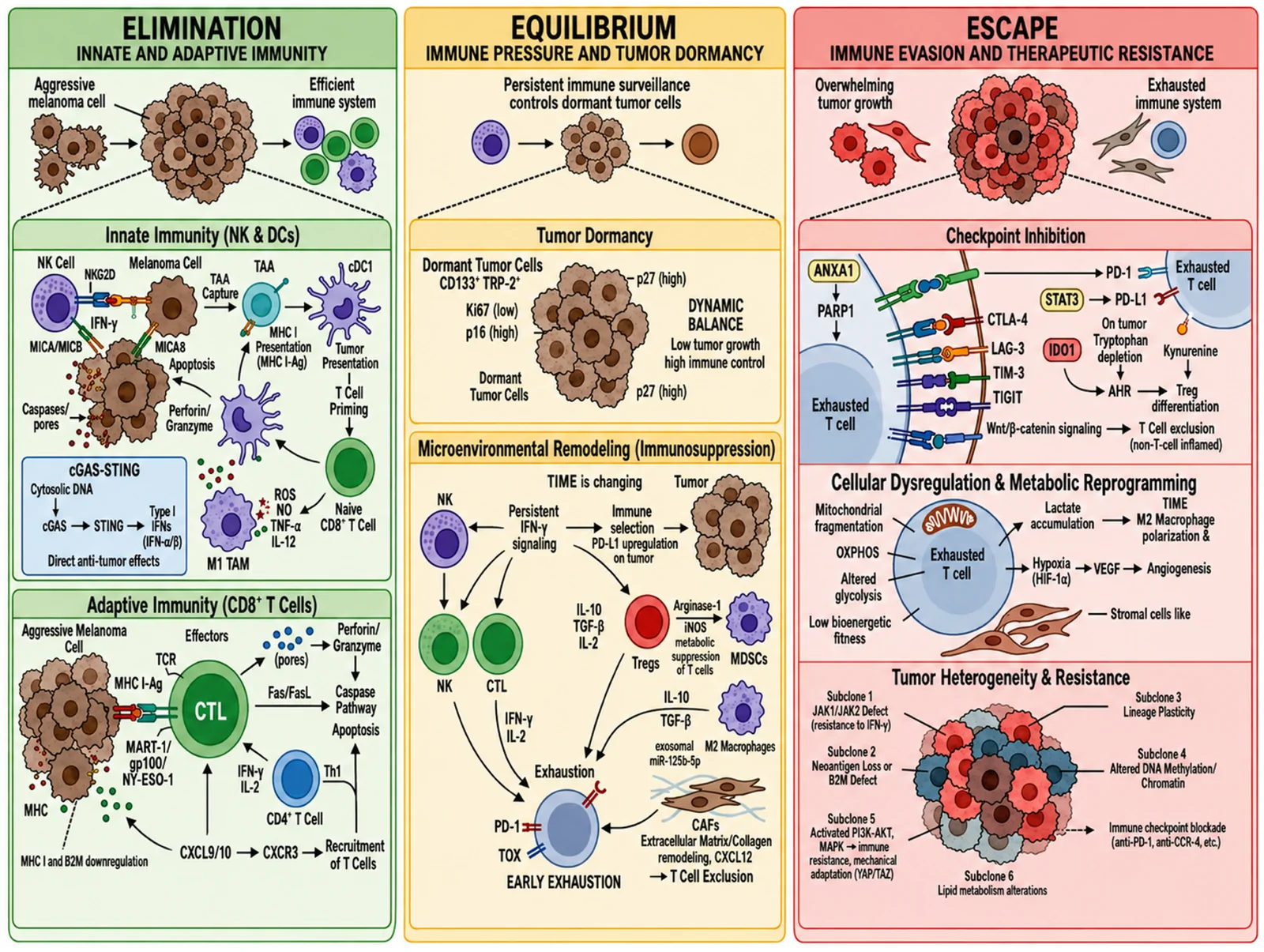
Cancer immunoediting in the melanoma tumor microenvironment.

## 4 Molecular mechanisms of immune escape in the melanoma microenvironment and therapeutic strategies

### 4.1 Key signaling pathways and molecular regulation of immune escape

During the immune escape phase, melanoma cells cooperate with multiple cellular components of the tumor microenvironment to suppress antitumor immunity through a range of signaling pathways and molecular mechanisms. Among these, the PD-1/PD-L1 axis represents one of the most critical immune checkpoint pathways. Melanoma cells and immunosuppressive cells within the TME, including myeloid-derived suppressor cells, can express high levels of PD-L1, which binds to PD-1 on T cells and promotes functional exhaustion while impairing T-cell survival and effector activity (57). PD-L1 expression is tightly regulated by signaling pathways such as JAK-STAT and NF-κB. For example, ANXA1 has been shown to interact with PARP1 and enhance STAT3 transcriptional activity, thereby promoting PD-L1 expression and facilitating tumor immune escape (58). In addition, emerging immune checkpoint molecules, including LAG-3, TIM-3, and TIGIT, are frequently co-expressed on exhausted T cells and further reinforce T-cell dysfunction (59). Accordingly, dual immune checkpoint blockade, such as combined targeting of PD-1 and LAG-3, has demonstrated clinically meaningful activity in melanoma. The indoleamine 2,3-dioxygenase 1 (IDO1)-mediated tryptophan metabolic pathway also contributes substantially to immune suppression during the escape phase. Tumor cells and myeloid cells can metabolize tryptophan into kynurenine through IDO1 activity, simultaneously depleting an amino acid required for T-cell function and generating immunosuppressive metabolites. Kynurenine can further activate the aryl hydrocarbon receptor (AhR), thereby promoting Treg differentiation and reinforcing immune tolerance (60). In parallel, constitutive activation of Wnt/β-catenin signaling is closely associated with T-cell exclusion in melanoma. Activation of this pathway can reduce the expression of chemokines required for dendritic-cell recruitment, thereby impairing the establishment of an inflamed tumor microenvironment and promoting a non-T-cell-inflamed immune-escape phenotype (61).

### 4.2 Functional exhaustion and metabolic reprogramming of immune cells in the tumor microenvironment

CD8^+^ T-cell exhaustion is one of the most characteristic forms of immune dysfunction during the escape phase. Exhausted T cells exhibit sustained expression of inhibitory receptors such as PD-1 and TIM-3, together with exhaustion-associated transcriptional and epigenetic programs driven by factors including TOX and members of the NR4A family. These changes progressively stabilize the exhausted state and are accompanied by reduced production of key effector cytokines such as IFN-γ and TNF-α (62, 63). As exhaustion becomes entrenched, the ability of T cells to proliferate, persist, and exert cytotoxic activity is markedly compromised. Mitochondrial dysfunction and metabolic reprogramming are major contributors to T-cell exhaustion. Exhausted T cells can display mitochondrial fragmentation, impaired oxidative phosphorylation, altered glycolytic activity, and reduced bioenergetic fitness, collectively limiting their capacity to sustain prolonged antitumor responses (64). At the same time, accumulation of lactate within the TME creates an additional metabolic barrier to effective immunity. Elevated lactate levels can impair T-cell signaling and cytokine production while promoting M2-like macrophage polarization and tumor angiogenesis (44, 65, 66). Hypoxia further amplifies this immunosuppressive environment. Stabilization of hypoxia-inducible factor 1α (HIF-1α) promotes angiogenic and adaptive responses in tumor cells and can enhance immune escape by increasing PD-L1 expression and suppressing NK-cell activity (67). Stromal and vascular-associated cells also contribute to metabolic and spatial immune exclusion. For example, in acral melanoma, a collagen-producing RGS5^+^/COL3A1^+^ perivascular cell population has been associated with vascular abnormalities and hypoxia. Increased abundance of this population correlates with reduced CD8^+^ T-cell infiltration and poorer responses to immunotherapy (68). These findings indicate that immune escape in melanoma is not solely driven by intrinsic T-cell dysfunction but also by spatial, metabolic, and stromal constraints imposed by the surrounding microenvironment.

### 4.3 Immunoediting-driven tumor heterogeneity and resistance to immunotherapy

Persistent immune selection during immunoediting favors the survival and expansion of tumor clones with reduced immunogenicity and enhanced resistance to immune-mediated elimination, thereby contributing to both primary and acquired resistance to immune checkpoint blockade. Defects in IFN-γ signaling, including alterations in JAK1 or JAK2, can reduce tumor-cell responsiveness to interferon-mediated growth inhibition and immune pressure and have been implicated in resistance to anti-PD-1 therapy (48, 69). Similarly, loss of tumor neoantigens or defects in MHC class I antigen presentation represent major mechanisms of acquired resistance. Genomic studies of resistant melanoma have identified truncating or loss-of-function alterations in β2-microglobulin, resulting in defective MHC class I surface expression and impaired recognition by CD8^+^ T cells (70). The heterogeneity generated by immunoediting extends beyond genomic alterations and includes differences in transcriptional states, epigenetic programs, and metabolic phenotypes. This multidimensional heterogeneity makes durable tumor control by single-target therapies particularly challenging (71, 72). For example, fatty acid metabolic reprogramming has been associated with distinct immune phenotypes, and molecular subtypes defined by lipid-metabolism-related features may have prognostic value and potential relevance to immunotherapy response (73). Immunoediting may also promote lineage plasticity. Under sustained immune and therapeutic pressure, subsets of melanoma cells can shift from differentiated melanocytic states toward mesenchymal-like or neural crest-like phenotypes, accompanied by loss of lineage-associated antigens and increased resistance to immune recognition (74, 75). Such phenotype switching provides melanoma cells with a non-genetic route to evade immune-mediated destruction. In addition, dysregulation of molecules such as YIF1B may contribute to remodeling of the TME, impairment of CD8^+^ T-cell function, and activation of inflammatory signaling programs, including the IL-6/JAK axis, thereby further promoting melanoma progression (76).

### 4.4 Combination therapeutic strategies based on the immunoediting framework

The immunoediting framework provides a rationale for developing stage-specific and mechanism-based combination therapies. During the elimination and equilibrium phases, immune adjuvants such as Toll-like receptor agonists or STING agonists may enhance dendritic-cell activation, antigen presentation, and type I interferon signaling, thereby increasing tumor immunogenicity and promoting conversion of immunologically “cold” tumors into more inflamed, immune-responsive states (77, 78). For tumors that have entered the escape phase, simultaneous targeting of multiple immunosuppressive pathways may be required. Potential strategies include combined blockade of PD-1/PD-L1 with additional checkpoint molecules such as LAG-3 or TIM-3 (79, 80). In parallel, metabolic interventions aimed at restoring T-cell fitness may complement immune checkpoint inhibition. Targeting lactate production or transport, mitochondrial dysfunction, or other metabolic constraints within the TME may help reverse metabolic exhaustion and improve antitumor T-cell activity (81–83). Epigenetic therapies also provide a potential means of reversing immunoediting-associated immune resistance. Histone deacetylase inhibitors and DNA methyltransferase inhibitors may restore the expression of silenced antigen-presentation machinery, interferon-responsive genes, and chemokines, thereby increasing tumor immunogenicity and potentially sensitizing melanoma cells to immunotherapy (54, 55, 84). Personalized neoantigen vaccines represent another attractive strategy. By identifying and targeting highly immunogenic mutations present within an individual tumor, these vaccines may broaden antitumor T-cell responses and help address the clonal heterogeneity generated through immunoediting (85). Finally, nanotechnology-based approaches may enable coordinated remodeling of the melanoma microenvironment and more precise delivery of immune-modulatory agents. For example, nanoparticle platforms have been explored for the delivery of gene-editing systems or agents targeting PD-L1, hypoxia, and other immunosuppressive features of the TME (86–88). Overall, combination therapies guided by the immunoediting state of individual tumors may provide a more effective strategy for overcoming immune escape and improving the durability of melanoma immunotherapy.

## 5 Conclusion

Tumor immunoediting provides a dynamic framework for understanding melanoma progression through the sequential phases of elimination, equilibrium, and escape. Within this process, T-cell exhaustion, accumulation of immunosuppressive cells, impaired antigen presentation, metabolic reprogramming, and lineage plasticity collectively promote immune escape and therapeutic resistance. Importantly, these mechanisms are not independent but interact to generate substantial spatial and temporal tumor heterogeneity, which contributes to both primary and acquired resistance to immunotherapy. Future therapeutic strategies should move beyond single-pathway intervention and incorporate the immunoediting status of individual tumors. Combination approaches integrating immune checkpoint blockade with metabolic modulation, epigenetic therapy, or personalized neoantigen vaccination may help restore antitumor immunity and overcome resistant tumor subclones. However, optimal treatment timing, patient stratification, and control of immune-related toxicity remain major challenges. Ultimately, translating tumor immunoediting from a conceptual model into a clinically applicable framework may improve patient selection, guide rational combination therapies, and advance precision immunotherapy for melanoma.
